# Acute Liver Failure Following Cytoreductive Surgery and Hyperthermic Intraperitoneal Chemotherapy: A Case Report

**DOI:** 10.7759/cureus.5026

**Published:** 2019-06-28

**Authors:** Pavneet Kohli, Prasanth Penumadu, Rajkumar Subramaniam.

**Affiliations:** 1 Surgical Oncology, Jawaharlal Institute of Postgraduate Medical Education and Research (JIPMER), Puducherry, IND

**Keywords:** cytoreductive surgery, pseudomyxoma peritonei, liver failure, hipec, hipec complications

## Abstract

Cytoreductive surgery (CRS) followed by hyperthermic intraperitoneal chemotherapy (HIPEC) is a commonly used one-step modality for peritoneal surface malignancies. Despite the gain in popularity and effectiveness, HIPEC is still associated with significant post-operative morbidity and mortality. Liver failure, following HIPEC surgery, which does not involve major liver resection has not been well defined or reported. Hence, we report a case of liver failure in a 51-year-old lady, with recurrent carcinoma ovary, who underwent cytoreductive surgery followed by HIPEC and the potential multifactorial causes that could have caused it.

## Introduction

The novel technique of cytoreductive surgery (CRS) was first described by Sugarbaker [1]. CRS along with hyperthermic intraperitoneal chemotherapy (HIPEC) is now a well-described modality for treatment of peritoneal carcinomatosis arising from gastrointestinal, gynaecological and primary peritoneal malignancies. Despite the increased use and better understanding of physiology associated with this unique surgical technique, the morbidity associated with it remains as high as 50% in certain series [2]. Intra-abdominal abscess, sepsis, pulmonary and renal complications are well described along with the increase in incidence due to the complexity, extent and duration of surgery [3]. Liver failure or hepatic insufficiency has not been described post CRS and HIPEC. Here, we describe a rare case of acute hepatic failure post CRS and HIPEC.

## Case presentation

A 51-year-old lady, with no known co-morbidities, was diagnosed with carcinoma ovary in May 2014. She underwent transabdominal hysterectomy with bilateral salpingo oophorectomy and infracolic omentectomy in June 2014. Histopathological examination (HPE) revealed a borderline mucinous tumour with invasive deposits in the right parametrium and omentum and features of pseudomyxoma peritonei. She received no adjuvant treatment in view of HPE showing pseudomyxoma. She was on regular follow-up and developed recurrence after a disease-free interval (DFI) of two years. A contrast enhanced CT (CECT) scan revealed omentum, splenic and liver sub-capsular deposits. She was treated with nine cycles of single agent Capecitabine. Post-chemotherapy, CECT scan revealed stable disease. She was subsequently referred to our centre for secondary cytoreduction. Her case was further evaluated, optimised, and discussed in a multidisciplinary tumour board and after optimisation was planned for surgery.

She underwent a diagnostic laparoscopy followed by cytoreductive surgery, which included anterior resection, right hemicolectomy, splenectomy, wedge excision of left lobe of liver, cholecystectomy and total peritonectomy with HIPEC. Intraoperative evaluation revealed multiple peritoneal deposits in the right and left sub-diaphragmatic regions. Multiple surface deposits were present over liver, spleen, rectum, sigmoid colon, right hemicolon, stomach, greater omentum, hepatoduodenal ligament, falciform ligament, epiploic foramen and ileum. Minimal mucinous ascites was present but there was no liver metastasis. Intraoperative assessment revealed a Peritoneal Cancer Index (PCI) of 22 (3 in region 0, 1, 2, 3 and 7; 2 in region 6, and 1 in region 4, 5, 8, 11 and 12). Intraoperative mitomycin (15 mg/m^2^) and adriamycin (15 mg/m^2^) were used and temperature was maintained at 42°C for 90 minutes. She received four units of packed cells, four units of platelets, and eight units of fresh frozen plasma (FFP) during the intra-operative period. Intra-operative period was uneventful.

On post-operative day 1, the patient developed jaundice. Biochemical analysis revealed a total bilirubin of 12.5 mg/dl (Direct 5.5), aspartate aminotransferase (AST) of 456 international units (IU), alanine aminotransferase (ALT) of 306 IU, and alkaline phosphate (ALP) of 28 IU. International normalised ratio (INR) was greater than 5.5 and prothrombin time was greater the 60 seconds. A Doppler ultrasound test revealed normal flow in the portal and hepatic veins as well as hepatic artery. Direct and indirect Coomb’s tests showed negative results. Peripheral smear revealed no evidence of haemolysis and urine analysis was also distinctly normal. There was no rise of total leukocyte count or fever. The patient was managed conservatively with FFP, multiple blood transfusions, 20% albumin infusion and Vitamin K. She responded to conservative management and gradually the hepatic parameters improved over the next 72 hours (Table 1). She was discharged from the hospital on post-operative day 20. She had regained her hepatic functions at the time of discharge.

**Table 1:** Progression of liver function tests in the immediate post-operative period. POD: Post-operative Day; AST: Aspartate Aminotransferase; ALT: Alanine Aminotransferase; ALP: Alkaline Phosphatase; PT: Prothrombin Time; INR: International Normalised Ratio.

Parameter	POD 1	POD 2	POD 3	POD 4	POD 6	POD 18
Total Bilirubin	12.5	4.95	4.29	2.85	2.43	0.89
Direct Bilirubin	5.54	1.86	2.38	1.49	1.19	0.46
Albumin	2.2	2.6	3.2	2.6	3.0	3.7
AST	456	345	167	83	52	71
ALT	306	203	158	103	61	61
ALP	28	33	60	67	89	156
PT	>60	19.5	15.3	13.7		13.7
INR	>5.5	1.51	1.15	1.01		1.01

## Discussion

Cytoreductive surgery and HIPEC involves complex surgical procedures and altered physiology and haemodynamics, which makes the post-operative care challenging. Gastrointestinal system complications during post HIPEC period ranges from 4 to 19%, with anastomotic leaks and fistulas being the most common ones [4,5]. Pleural effusion forms the most common pulmonary complication (10%). Haematological complications include neutropenia and thrombocytopenia with incidence of it varying because of the practice heterogeneity in the drug used, its dosage, duration and temperature [6]. Other well-defined complications include renal insufficiency (2-4%), venous thromboembolism (4-4.4%), urinary tract infections and vascular access infections [7,8].

A review of literature revealed only two cases of severe haemorrhagic shock combined with hepatic insufficiency and necrosis following HIPEC using oxaliplatin on post-operative day 3 and post-operative day 1, respectively [9]. The authors in their report document that histopathological analysis of necrotic hepatic tissues did not reveal the cause of injury. Hepatic parenchyma was difficult to identify and the specimens mostly composed of blood clots and devitalized necrotic tissues. As mentioned, hepatic insufficiency post CRS and HIPEC is not well described in the literature.

Mitomycin is a cytotoxic antibiotic first isolated from *Streptococcus caespitosus* and later shown to have potent antitumor effects in vitro and in vivo. Side effects of mitomycin include bone marrow suppression, nausea, vomiting, diarrhoea, stomatitis, rash, fever and malaise. Rare complications include haemolytic uremic syndrome, haemolysis, neurological abnormalities, renal failure and interstitial pneumonitis [10]. Use of mitomycin with other chemotherapy drugs has been shown to have mild and transient elevation of liver enzymes which resolves spontaneously and as such it is difficult to attribute this elevation to mitomycin alone [11]. High doses of mitomycin have been linked to cases of sinusoidal obstruction syndrome but these typically present 10 to 30 days after the infusion [12]. Hence, we believe that systemic toxicity due to mitomycin might be an unlikely cause of hepatic insufficiency.

Adriamycin is an anthracycline antibiotic which acts by intercalating between DNA base pairs and uncoiling the DNA helix. Serum aminotransferase elevations occur in up to 40% of patients but elevations are symptomatic and generally transient. Liver toxicity with elevated bilirubin levels and deranged coagulopathy are rare. The toxicity is a result of production of a toxin or immunogenic intermediate which causes direct liver toxicity [13].

Entrapment of mitomycin or adriamycin between the Glisson’s capsule and hepatic lacerations could be another cause for continued effect of mitomycin, as a result it causes local toxicity and hepatocyte necrosis. Mitomycin can cause sinusoidal obstruction which is unlikely, with possibility of chemo-induced vasculitis [12]. An atypical hypersensitivity reaction could be another possible cause.

HIPEC is a surgery unlike any other and involves hyperthermia with temperature going up to 42°C. In our practice, we applied a plateau pressure of 42°C for 90 minutes for all patients. However, none of our other cases have reported any such thermal complications. It is possible that hepatic thermal injury was induced by the inflow pump placed over the liver for a long time or a misplaced heat generator which might have resulted in hepatocyte necrosis secondary to heat.

A Glisson capsulectomy of liver is warranted if deposits are present over the liver. We used mono polar diathermy to achieve this dissection. In vitro studies in pig liver have demonstrated that monopolar coagulation takes 33% more time for coagulation and haemostasis than bipolar coagulation technique. Longer coagulation time results in more stickiness of the necrotic tissue to the instrument. This leads to new bleeding points and extends the coagulation time. The average tissue loss was 6.0 mm in monopolar technique and 4.5 mm in bipolar [14]. The temperatures generated at the tip of the instruments varied considerably with instrument type, power setting and length of time of application. The highest mean temperature (standard deviation) recorded at the tip of monopolar and bipolar diathermy can range up to 78.9 (4.1) and 41.9°C (2.2), respectively. Application of monopolar diathermy (10 seconds at 40 W) resulted in a mean (standard deviation) temperature of 59.2°C (2.2) in tissues 1.0 cm away from the instrument tip [15]. The use of monopolar diathermy generates the highest temperatures and results in marked lateral thermal spread, which might be one of the major causes for elevated liver parameters in our patient.

Haemolysis due to multiple blood transfusions can also elevate liver enzymes but was ruled out by a negative Coomb’s test.

The clinical syndrome of “shock liver”, a form of ischemic hepatitis, which is characterised by elevation of liver enzymes to up to 20 times the normal as a result of systemic hypotension, was also considered as a differential point [16]. However, a review of the intraoperative anaesthesia notes revealed no transient or prolonged hypotension.

## Conclusions

To conclude, hepatic insufficiency is an unusual yet potentially devastating complication of HIPEC. Etiology can be multifactorial. In our case, we could not pinpoint one causative factor. Thermal injury could be a major cause reason, and therefore, extreme caution should be exerted to avoid it.
